# Chicago Medical Society

**Published:** 1869-06-01

**Authors:** 


### *Proceedings of the Chicago Medical Society.*

FRIDAY EVENING, April 30th, 1869.

Regular weekly meeting of the society, President Bogue in the chair. Exhibition of pathological specimens being in order, Vice President Schmidt presented several pieces of comminuted bone, from the elbow-joint of a man. The fracture involved the condyles of the humerus as also the head of the radius and olécranon of the ulna. Resection of the bones was accomplished with the chain saws, eighteen months since. The patient, a musician, has regained good use of his arm. The doctor thinks where the olecranon or other bony prominences are left in the wound, the cure is retarded.

Dr. Wanzer reported a case of fracture of the external condyle of the humerus, and a part of the shaft; that after removing the comminuted bones, the limb was dressed at right angles; that active inflammation supervened; that so great was the sensibility of the part and irritability of the patient, that passive motion was prevented without anæsthesia; that bony anchylosis was the result, but the patient has very good use of his arm.

Dr. Danforth asked if we ought not to expect from surgery better results than anchylosis in such cases.

Dr. Bogue thinks anæsthetics are indicated that with them occasional passive motion should be attempted, to prevent permanent anchylosi s.

Dr. Loverin thinks the rectangular position correct; that where such extreme sensibility of the part exists, that passive motion need not be persisted in.

Dr. Wanzer reported a case of hydrocele involving the left half of the scrotum. The patient had been suffering five years until its size became inconvenient, and the traction upon the cord was very painful; a quart of liquid was evacuated with the canula, and a seton passed through the aperture and above. The parts have contracted to nearly their normal size, and we hope the cure is radical.

Dr. Toverein is in favor of iodine injected into the sac; has seen adhesive inflammation and contraction follow.

Dr. Bogue stated he had used the iodine with occasional benefit, but had found operation by incision always a radical cure. His method is to lay the sac freely open.

Dr. Wanzer reported a case of double hydrocele, which he had tapped several times, and was afterward cured radically by incision.

On motion, Society adjourned.

---

CHICAGO, May 7, 1869.

Regular weekly meeting of the society, President Bogue in the chair.

The discussion for the evening being in order—What is the pathology and best treatment for cystitis, both acute and chronic?

Dr. Quales stated that the causes most frequent were traumatic, viz.: by the maladroit use of instruments, blisters, hemorrhoids, and foreign bodies in the bladder, stricture urethra, or fistula in ano. It may be acute or chronic. The treatment in the acute form should be more local than general, viz.: warm baths, enemas, and the recumbent posture. In the chronic stage, bal. copaiba, the terebinthinates, and decoctions buchu. The diet should be light in the acute, but more supporting in the chronic variety.

Dr. Paoli stated that there were few practitioners of ten years' standing but had seen cystitis; that the causes of the acute form are often cold, with those who work in low, damp localities; the causes of the chronic were gravel, stricture urethra, and fistula; that the disease was often overlooked. The symptoms are tenacious mucus in the urine, painful micturition, pain over the pubis, and general debility. He recommends decoction uva ursi and injections of tepid water into the bladder.

Dr. Reid said his experience in the traumatic variety was limited, but has treated many cases of the chronic variety; reported a case in an old lady, who passed much tenacious mucus. There was frequent micturition, urine often bloody. She was much debilitated, but finally recovered; that she afterward relapsed in consequence of intemperance, and the doctor withdrew from the case. He treated her first with bal. copaiba and black drop, and found benefit from decoction uva ursi. Where there were bloody discharges he used the terebinthinates, with tinct. opii; had resorted to ferruginous tonics, laxatives, anodynes, suppositories, injections of solution plumbi acetas into the bladder.

Dr. Danforth stated that, in the acute form, his treatment was both local and general. Locally, the hot bath, fomentations of hops and opium over the bowels; internally, linseed tea, the watery solution of opium and belladonna, with occasional saline purgatives. In the chronic form, had used ergot. Where the discharges were pus and mucus, used acid nitric, with opiates and vescical injections of argent. nitras, grs. 2 to 1 pint water. Had used tonics and stimulants, and had found the disease very obstinate.

Dr. Bogue stated he had found in acute cystites the hot hip bath beneficial; also washing out the bladder with tepid water, suppositories of morphia and belladona into the rectum, with fomentations over the bowels beneficial. The bowels should be kept soluble; in the chronic variety thinks the hypertrophy and inflammatory condition not always permanently relieved, and the disease is liable to return. He says the measures he adopts are injections in the bladder of weak solution argent. nitras, cupri sulphas or zinci. The causes of the disease are stone or other foreign bodies in the bladder; thinks radical cure may be accomplished by opening the bladder, allowing it to be at rest; says cystitis in old people is generally caused by enlarged prostate. In such cases the catheter is the remedy with injections of tepid water into the bladder. Where stricture-urethra is the cause to relieve the stricture.

Dr. Paoli stated that it was difficult to introduce the catheter in old people, where the disease had lasted many years in consequence of hemorrhage, and that the coats of the bladder had degenerated.

The Society then adjourned.

Hiram Wanzer, Sec'y.

